# Neuromorphic Systems Design by Matching Inductive Biases to Hardware Constraints

**DOI:** 10.3389/fnins.2020.00437

**Published:** 2020-05-28

**Authors:** Lorenz K. Muller, Pascal Stark, Bert Jan Offrein, Stefan Abel

**Affiliations:** Neuromorphic Devices and Systems Group, Science and Technology Department, IBM Research Zurich, Rüschlikon, Switzerland

**Keywords:** neural network, neuromorphic, bias, constraint, inductive bias, sparsity, regularization, collaborative filtering

## Abstract

Neuromorphic systems are designed with careful consideration of the physical properties of the computational substrate they use. Neuromorphic engineers often exploit physical phenomena to directly implement a desired functionality, enabled by “the isomorphism between physical processes in different media” (Douglas et al., 1995). This bottom-up design methodology could be described as matching computational primitives to physical phenomena. In this paper, we propose a top-down counterpart to the bottom-up approach to neuromorphic design. Our top-down approach, termed “bias matching,” is to match the inductive biases required in a learning system to the hardware constraints of its implementation; a well-known example is enforcing translation equivariance in a neural network by tying weights (replacing vector-matrix multiplications with convolutions), which reduces memory requirements. We give numerous examples from the literature and explain how they can be understood from this perspective. Furthermore, we propose novel network designs based on this approach in the context of collaborative filtering. Our simulation results underline our central conclusions: additional hardware constraints can improve the predictions of a Machine Learning system, and understanding the inductive biases that underlie these performance gains can be useful in finding applications for a given constraint.

## 1. Introduction

A variety of systems are referred to as “neuromorphic,” Originally, “neuromorphic” has referred to the idea of making use of isomorphisms between physical processes in different media, for example, drift-diffusion phenomena in silicon to emulate drift-diffusion in neuronal ion channels, in order to build VLSI chips consisting of neuron-like elements (Mead, 1989; Douglas et al., 1995; Indiveri et al., 2011). Now, the term is used more broadly and also encompasses systems that accelerate artificial neural network (ANN) algorithms (Hu et al., 2016) or use a biomimetic processing principle (Furber, 2016).

Most neuromorphic systems have in common that parameters implemented in them or in the larger system around them are learned from examples. If this learning process should generalize to unseen examples, it is well-known that it needs to be biased in some way. Such biases that help a learning system generalize from its training data are known as inductive biases (Mitchell, 1980).

**Figure 1:**
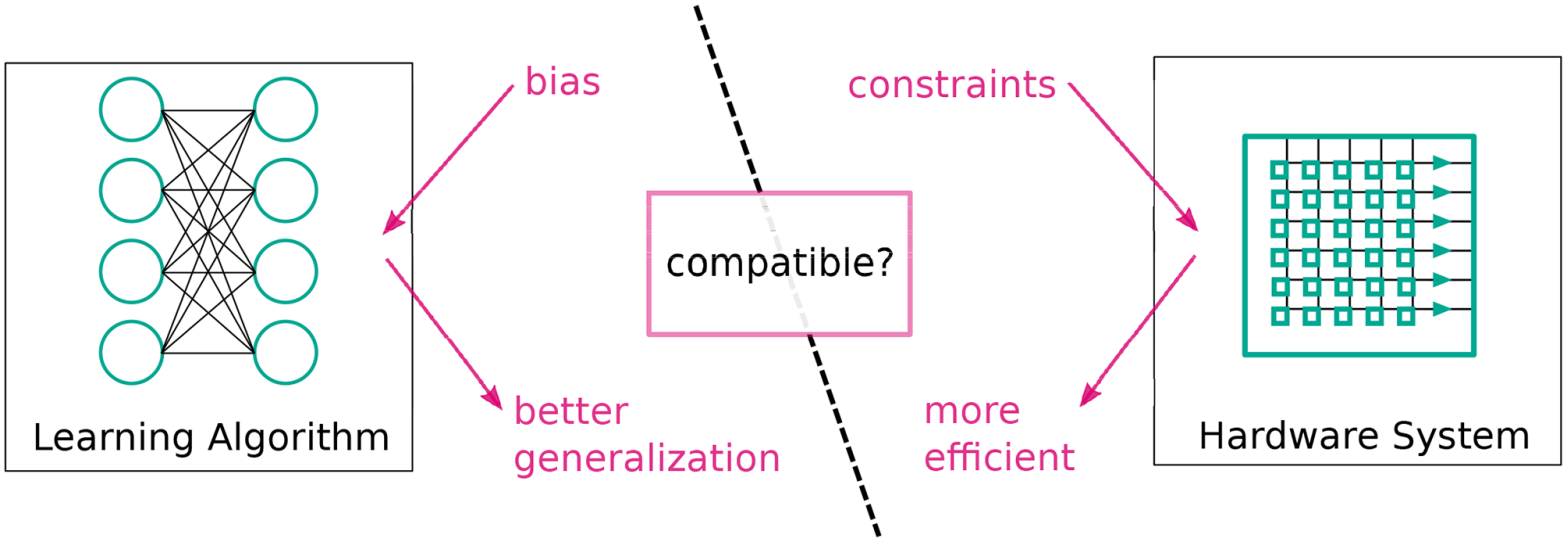
General concept of bias matching: we should try to match inductive biases of learning algorithms to constraints on hardware to obtain systems that both generalize better and are more efficient.

From an algorithmic perspective, inductive biases can come in many forms: the algorithm's structure, i.e., how parameters affect the output (Tai et al., 2015), regularization, e.g., additional costs (Krogh and Hertz, 1992), constraints on parameters during training (Ioffe and Szegedy, 2015), or, in the case of Bayesian models, explicitly as priors such as those described by Griffiths (2010). These concepts can also be interpreted as constraints on a learning algorithm's complexity. From this point of view, it is evident that, in an ideal world, the hardware on which the algorithm is implemented exploits this simplicity for more efficient processing (see Figure 1).

In this paper, we have asked two questions: “Can additional hardware constraints improve the predictions of a Machine Learning system?” and “What inductive biases underlie these performance gains?” We answered these questions by giving concrete examples and new conceptual designs of “bias matching”: hardware implementations of machine learning algorithms, where a useful inductive bias can be exploited for efficient computation. Our aim was to establish “bias matching” as a high-level approach in the design of neuromorphic hardware.

“Bias matching” contrasts, as a design-philosophy, with a traditional bottom-up approach to neuromorphic engineering, e.g., as described by Douglas et al. (1995). In the bottom-up approach “the efficiency […] rests in the power of analogy, the isomorphism between physical processes in different media” and “computational primitives such as conservation of charge, amplification, exponentiation, thresholding, compression and integration arise naturally out of the physical processes of aVLSI circuits.” In this bottom-up approach the focus is on computational primitives and their efficient implementation, whereas we focus on inductive-biases and how to exploit them for efficiency.

We will discuss our approach based on examples concerning the following inductive biases/hardware constraints and elaborate why they may be relevant for hardware design (see also Table 1 for an overview):

1. Translation and time-shift equivariance (section 2.1)
2. Spatio-temporal locality (section 2.2)
3. Frequency limitations of input signals (section 2.3)
4. Sparse, low-rank and kernelized low-rank connectivity (sections 2.4, 2.5 4.1)
5. Low-resolution connection weights (section 2.7)
6. Regularization by batch-size choice (sections 2.6, 4.2).

**Table 1:** An overview of the pairings of hardware constraints and inductive biases discussed in this paper.

**Hardware constraint**	**Inductive bias**	**Application area**	**Section**
Tied weights between neighborhoods	Translation/Shift equivariance	Spatial and/or temporal signals, e.g., images, audio, video	2.1
Local communication/decaying memory	Local independence, spatially/temporally hierarchical models	Spatial and/or temporal signals, e.g., images, audio, video	2.2
Slow state change	Eigenvalues of recurrent network closer to one	Speech processing	2.3
Connectivity/memory limitations	Sparse and low-rank connectivity	Collaborative filtering, model compression	2.4, 4.1
Low-resolution weights	Difficult to interpret, possibly anti-synergistic with SGD	Unknown	2.7
No gradient aggregation over samples	Batch-Size regularization	Collaborative filtering (among others)	2.6, 4.2

For each of these, we have defined an inductive bias or hardware constraint, given (where possible) an example of its relevance, and outlined how it can impact design. We have looked at hardware and software implementations from the literature (section 2), and we have presented novel observations and simulations to back up our claims (section 4).

## 2. Background

In this section, we have examined examples of neural network implementations from the literature through the lens of the “bias matching” design perspective we propose in this paper.

### 2.1. Translation and Time-Shift Equivariance by Tying Weights

#### 2.1.1. Inductive Bias

Probably the best know examples of an inductive bias in the context of neural networks are convolutional neural networks (CNNs) (Fukushima, 1988; LeCun et al., 1995) that exploit translation equivariance. Translation equivariance (e.g., Worrall et al., 2017), means that, if we translate the input of our model, its output remains the same up to a translation. On the example of images and CNNs, we can formulate translation equivariance: given a CNN layer *L*(·), image *X*, and the translation operator *T*, there exists an operator *t* such that

(1) $$\begin{array}{r} L\left(T\left(X\right)\right)=t\left(L\left(X\right)\right) \end{array}$$

Concretely, for a stride-1 convolution with appropriate padding, *T* and *t* are the same and *T*(*X*_*i, j*_) = *t*(*X*_*i, j*_) = *X*_*i*+*t, j*+*s*_, where *i* and *j* are pixel indices, and *s, t* are small natural numbers. Notably, full CNNs are not translation equivariant, but single convolutional layers are.

CNNs achieve equivariance by enforcing that some of their parameters are equal (often referred to as “tying” parameters). A given neuron *n* in a CNN receives input from some window *w* of the previous layer's output. For every other window *w*′ there exists by construction a neuron *n*′ with the same input weights as *n* (these weights are however applied to a different input, namely *w*′). In this sense, the weights of some sets of neurons in a CNN are tied together (not independent).

Note that time-shift equivariance is a special case of translation equivariance in one dimension.

#### 2.1.2. Hardware Constraint

Because of its implementation in the form of weight tying, equivariance is highly relevant for hardware implementations of CNNs. All neurons belonging to the same input/output channel pair have the same weight. Hardware implementations of CNNs making use of this constraint, holding each weight in their memory only once and applying them to different sections of the input either by broadcasting (Bose et al., 2019) or sequentially, as, for example, on GPUs (Chetlur et al., 2014). On GPUs, the convolution operation is commonly recast as a highly optimized general matrix multiple between the filters and a copied and tiled input image (though many variants of GPU convolutions exist).

Recurrent neural networks (RNNs) keep their weights constant between subsequent time steps and implement time-shift equivariance in this way. Due to the sequential and non-linear nature of RNNs, implementations necessarily operate in a fixed time-unrolled order and explicitly implement weight tying.

#### 2.1.3. Performance Impact

Weight-tied neural networks are state-of-the-art in many applications, particularly in the audio-visual domain. Some highly cited examples include digit recognition (LeCun et al., 1989), image classification (Cireşan et al., 2011; Krizhevsky et al., 2012; He et al., 2016), and speech recognition (Saon et al., 2017).

### 2.2. Spatial and Temporal Locality by Neighborhood Communication

#### 2.2.1. Inductive Bias

Spatial locality means that, in order to compute a quantity of interest at a given point *p*, only points that lie in a small neighborhood of *p* need to be taken into account simultaneously. Spatially localized operations are often used to build a hierarchy of features with increasing spatial extent. The inductive bias associated with spatial locality is therefore either directly the independence of neighborhoods or the breaking down of concepts into a spatial hierarchy.

Temporal locality occurs commonly in Reservoir Computing (RC) (Lukoševičius et al., 2012) because most RC systems are designed such that the echo-state property (ESP) holds. Formally, the ESP states that the influence of any input signal vanishes asymptotically (Jaeger, 2007). RC is particularly effective when temporally local information is sufficient to solve the given task.

#### 2.2.2. Hardware Constraint

Spatial locality is a key reason why CNNs can be computed efficiently (next to translation equivariance). The computation performed by neurons in a CNN is spatially localized if they have a small associated filter. An example of a hardware implementation of spatially localized processing is the SCAMP-5 sensor/processor array (Carey et al., 2013). The nearest-neighbor communication structure of this chip allows for an efficient pixel-parallel implementation of convolution filters if the filters are small (Bose et al., 2019). In GPU implementations of CNNs, small filters need fewer replications of each source pixel (as well as less memory for the filters themselves).

A class of hardware implementations that benefit from temporal locality are photonics-based RC, like Vandoorne et al. (2014). For silicon-photonics based systems, the integration of photonic amplifiers can be challenging, making temporal locality desirable. However, the operation of time-shifted addition (with small time shifts) is very efficient in these systems, allowing for cheap communication across “temporal neighborhoods.”

#### 2.2.3. Performance Impact

For spatial locality and associated CNNs, see section 2.1.2.

An example of a very high through-put system made possible by temporal locality implemented in photonic hardware is Larger et al. (2017).

### 2.3. Low-Frequency Signal Components and Slow Neurons

#### 2.3.1. Inductive Bias

For signals with slow dynamics (opposite to temporally local signals), an opposite approach can be useful. When analyzing signals, some of whose salient dynamics are much slower than the sampling rate, it can be difficult to learn effective weights for recurrent neural networks (RNN) because longer time dependencies are more difficult to discover. A commonly used remedy for this is low-pass filtering of the hidden state of the RNN (Mozer, 1992). This inductive bias could also be described as enforcing eigenvalues of the recurrent connection matrix that are closer to one (Nair and Indiveri, 2019a) in a linearized approximation of the RNN.

#### 2.3.2. Hardware Constraint

In a physical implementation, the fact that states of hidden neurons change slowly can be exploited by implementing them as leaky-integrate-and-fire (LIF) neurons with spike-frequency adaptation, which need to emit only few spikes to represent their state (Nair and Indiveri, 2019b). From the electrical engineering perspective, such neurons can be interpreted as ΣΔ-Modulators with unsigned Δ steps (Yoon, 2016).

#### 2.3.3. Performance Impact

Nair and Indiveri (2019a,b) indeed observed that, when the time-constant of such neurons matches the salient structure of the analyzed signal (i.e., a favorable inductive bias in the sense of Mozer, 1992 is used), the resulting system exceeds the performance of an unconstrained system while operating at very low power.

### 2.4. Linear Low-Rank Matrix Approximation by Parameter Sharing

#### 2.4.1. Inductive Bias

Strict low-rank matrix approximations (Koren et al., 2009) model a *n* × *m* matrix *W* as *W* = *QR*, where *Q* is *n* × *k* and *R* is *k* × *m*, where *k* is the resulting rank of *W*. Equivalently we can write the entries of *W* as dot-products of rows and columns of *Q* and *R* respectively:

(2) $$\begin{array}{r} w_{ij}={\vec{q}}_{i}\cdot {\vec{r}}_{j}. \end{array}$$

Low-rank matrix approximations are commonly used to model very large matrices from sparse observations, for example, in collaborative filtering (Koren et al., 2009).

#### 2.4.2. Hardware Constraint

Low-rank approximation of connection matrices in neural networks is straightforward to implement with efficiency gains on general matrix multipliers (GEMMs). This is because a connection matrix restricted to rank-k is equivalent to the interposition of a size-k layer with a linear activation function. In formulae, we can write for a neural network layer with an *n* × *m* weight matrix *W* that is rank-*k*; it can be written as *W* = *QR*, where *Q* is *n* × *k* and *R* is *k* × *m*:

(3) $$\begin{array}{r} W\vec{x}=QR\vec{x}=Q\left(R\vec{x}\right)=Q\vec{y}. \end{array}$$

The time complexity of this moves from *O*(*mn*) for $W\vec{x}$ to *O*(*k*(*m* + *n*)) for performing $R\vec{x}$ followed by $Q\vec{y}$. The memory required to store the parameters of *W* or *R* and *Q* respectively also scale this way: the individual entries of the matrix *W* share parameters.

#### 2.4.3. Performance Impact

Low-rank reparameterization has been proposed as a model compression tool for neural networks, both fully-connected (Denil et al., 2013; Sainath et al., 2013) and convolutional ones (Jaderberg et al., 2014). Recent examples of practical efficiency tweaks that can be interpreted as low-rank approximation are the linear bottleneck and depth-wise convolutions of Mobile-Net-v2 (Sandler et al., 2018) (note that depth-wise convolutions may additionally interpose a non-linearity).

### 2.5. Kernelized Low-Rank Matrix Approximation by Parameter Sharing

#### 2.5.1. Inductive Bias

Kernelized matrix reparameterization (Liu et al., 2016) generalizes the dot-product to any kernel function:

(4) $$\begin{array}{r} w_{ij}=K\left({\vec{q}}_{i},{\vec{r}}_{j}\right) \end{array}$$

It has been shown that such kernelized reparameterizations can impose interpretable structure on neural networks (Muller et al., 2018).

#### 2.5.2. Hardware Constraint and Performance Impact

Kernelized reparameterizations are more complicated to implement directly, and, to the best of our knowledge, this has not been discussed in the literature. The same reformulation as above does not work because the analog of $\vec{y}$ would live in the embedding space of the kernel function, which can be infinitely dimensional. However, kernelized reparameterizations have greater representational power than strict low-rank approximations and have been shown to produce state-of-the-art results in collaborative filtering benchmarks (Muller et al., 2018). In the case of architectures where the limiting factor is memory access, kernelized reparameterizations can also be associated to a speed-up: instead of looking up the *nm* entries of *W*, they can be computed from only *k*(*n* + *m*) values (entries of the matrix *W* share parameters). The additional overhead is the evaluation of a kernel function.

### 2.6. Batch-Size Regularization With Model Parallelism

#### 2.6.1. Inductive Bias

Standard neural network training by stochastic gradient descent (SGD) and its variants can be seen as a kind of regularization or inductive bias in itself (Neyshabur et al., 2017) (SGD with a small learning rate, is more likely to find a solution with small parameter values). Furthermore, in the case of mini-batch gradient descent (where gradients are summed or averaged over a “mini-batch” of examples before being applied to weights), decreasing the batch-size is often associated with better generalization (Wilson and Martinez, 2003). This may, however, depend on the exact variant of gradient descent used (Smith, 2018).

#### 2.6.2. Hardware Constraint and Performance Impact

For standard GPU implementations of neural networks, this is somewhat problematic because parallelization is most easily implemented as data paralellism over the batch dimension (Chetlur et al., 2014; Krizhevsky, 2014). In the worst case, this results in a trade-off between speed-up and generalization performance. In contrast, hardware implementations with weight-wise parallelism in the vein of Gokmen and Vlasov (2016) can have difficulties aggregating gradients over multiple samples but do not have to make the speed-generalization trade-off.

### 2.7. Low Resolution Synaptic Weights

#### 2.7.1. Inductive Bias

To the best of our knowledge, there is no clear inductive bias associated with the use of low-resolution synaptic weights, and, consequently, it is unclear what task or learning setup matches low resolution constraints. Intuitively, low resolution arithmetic might not match the setting of gradient-based training because the gradient only gives reliable information in a small neighborhood around the current model parameters'circumstantial evidence for this is the significant amount of work on the improvement of training methods in the context of low-resolution weights (e.g., Müller et al., 2017; Alizadeh et al., 2019; Helwegen et al., 2019). More generally, Goodfellow et al. (2014) argue that current neural network architectures are selected under the constraint that they are well-suited for training by SGD. The improvement of alternative training methods (e.g., gradient-free ones) could, in light of this, be impactful for low resolution neural networks.

#### 2.7.2. Hardware Constraint

In digital hardware, lower resolution directly translates into more compact designs. In analog hardware, there probably is an analogous trend due to noise tolerance. The optimal implementation of the low-resolution arithmetic for neural networks is in itself an open research question. Both floating- (Courbariaux et al., 2014) and fixed-point (Lin et al., 2016) approaches exist combined with different number formats (Langroudi et al., 2019) and compression approaches (Aimar et al., 2018). For the extreme case of binary and ternary weights (Courbariaux et al., 2015; Muller and Indiveri, 2015), the multiplication between inputs and synaptic weights can also be simplified, as in Courbariaux et al. (2015) or by sparse versions thereof.

#### 2.7.3. Performance Impact

State-of-the-art neural networks, in terms of pure predictive power, use at least 16-bit floating point arithmetic in all applications we are aware of. However, some ultra-low-resolution systems are highly competitive in terms of performance per power (Andri et al., 2016) or performance under limited memory usage (Uhlich et al., 2020).

## 3. Methods

In this section, have given implementation details of the simulations in the following section. We limited ourselves to dense, technical descriptions here and have given more context in the following section. All models were implemented in tensorflow (Abadi et al., 2016).

### 3.1. Bias Matching

In section 4, we have given two examples of how to apply bias matching in a concrete situation. Here, we have provided an abstract step-by-step description of bias matching.

1. Define a hardware property or constraint.
2. Define an end-to-end machine learning architecture incorporating the given constraint.
3. Find tasks that benefit from inductive biases associated with the constraint.

If necessary, revisit point two after the evaluating performances. While we followed this series of steps in the examples, one could also take an inductive bias as the starting point and work toward a hardware constraint.

### 3.2. Sparse Connectivity With Recurrent Fixed Weights

In this section, we have defined a neural network layer whose performance we have compared to that of a standard fully-connected layer in two different settings.

The layer we proposed, termed the sparseRec-layer, has the following recurrent definition (the reasoning behind this definition is given in section 4.1), given input $\vec{x}$:

(5) $$\begin{array}{l} {\vec{y}}_{0}=W_{in}\vec{x}\\ {\vec{y}}_{t}=f\left({\vec{y}}_{0}^{*}+W_{rec}{\vec{y}}_{t-1}\right) \end{array}$$

where *W*_*in*_ is a learned input *n* × *k* matrix, and *W*_*rec*_ is a fixed, randomly drawn recurrent *m* × *m* connection matrix. We chose *m* > *k* and will denote $s=\frac{k}{m}$ as sparsity. ${\vec{y}}_{0}^{*}$ is ${\vec{y}}_{0}$ zero-padded from length *k* to *m*. *f*(·) is an activation function. When computing the output of such a layer, we applied this recurrent definition up to *t*_max_ while keeping the input fixed.

#### 3.2.1. MNIST

The baseline model is a multilayer perceptron with one hidden layer trained on MNIST (LeCun et al., 1998). The hidden layer has *m* ∈ {16, 31, 62, 125, 250, 500} neurons and a rectified-linear activation function (Glorot et al., 2011). We trained with the Adam optimizer (Kingma and Ba, 2014) for 40 epochs at a batch size of 256 and summed categorical cross-entropy cost. We used drop-out regularization (Srivastava, 2013). We ran a hyperparameter sweep for dropout values *d* ∈ {0.0, 0.2, 0.4, and 0.6} and learning rates *l* ∈ {0.0005, 0.001, 0.002, and 0.003} with five different random seeds. We selected the best performing parameters on a validation set and report the best average performance of each model.

Formulaically the networks prediction given input $\vec{x}$ is

(6) $$\begin{array}{r} y=\text{softmax}\left(W_{\text{out}}\cdot \text{Dropout}\left(\text{ReLU}\left(W_{\text{in}}\cdot \text{Dropout}\left(\vec{x}\right)\right)\right)\right) \end{array}$$

The sparseRec model is identical with some changes: the hidden layer is replaced with a sparseRec-layer, as described in Equation (5), and also has a rectified-linear activation function. The sparsity s and the corresponding number of non-zero columns k of the feedforward matrix *W*_*in*_ is given in Table 2. The values of *W*_*in*_ are constrained to lie in [−1, 1] by reprojection after each optimization step. The recurrent matrix *W*_*rec*_ is set to fixed uniformly random weights of density 0.2 and rescaled to have spectral radius 0.95 (motivated by the echo-state property, this limits gradient decay/explosion).

**Table 2:** Basic network parameters used in the simulations in section 3.2.

**# hidden units (m)**	**# connected hidden units (k)**	**Sparsity (s) (%)**
500	500	0
500	250	50
500	125	75
500	62	87.6
500	32	93.2
500	16	96.8

Our goal was to compare the predictive accuracy of the two models as a function of the number of free parameters.

#### 3.2.2. ML1M

The baseline model is an item-based autoencoder identical to the one described in Sedhain et al. (2015). It has one or two hidden layers with *m* ∈ {280, 300, 350, 400, 450, 500} neurons (each) and sigmoid activation function. We optimized using full batches and the L-BFGS optimizer (Zhu et al., 1997) on the summed squared error of known entries with an L2 regularization strength *l*_2_ ∈ {25, 50, 100}. The L2 regularization was applied to the connection weights (not to biases) in the form of a cost $c=l_{2}\underset{ij}{\sum }{\left(W_{ij}\right)}^{2}$.

The sparseRec model is identical with the some changes: the hidden layer is replaced with a sparseRec-layer, as described in Equation (5), with a sigmoid activation function (as in the baseline model). The sparsity s and the corresponding number of non-zero columns k of the feedforward matrix *W*_*in*_ is given in Table 2. The values of *W*_*in*_ are constrained to lie in [−1, 1] by reprojection during the optimization. The recurrent matrix *W*_*rec*_ was set to fixed uniformly random weights of density 0.2 and rescaled to have spectral radius 0.95.

As for the MNIST dataset, we compared the predictive accuracy of the two models.

### 3.3. Batch-Size Regularization in Low-Rank Matrix Approximation

The model used was an Factorization Machine (FM) as described in Rendle (2012), where we adopted two minor deviations from this description also used in the code accompanying that paper: weight-decay (L2 regularization) was only applied to parameters that have non-zero gradient, and the models output was restricted to the range of the rating values given in the training set. Finally, we added a modification for numerical stability with large batch sizes: the gradient of the global bias *b* was divided by the batch-size.

For each batch-size, we individually found the optimal hyperparameters (L2 regularization strength *l*_2_, learning rate) in {0.02, 0.04, 0.06, 0.08} × {0.0005, 0.001, 0.002, and 0.003}. *l*_2_ is the multiplicative coefficient to an L2 cost given in the previous subsection. We ran each model for at least five different random seeds (resulting in different initial parameters and different train-test splits). For each batch-size, we picked the hyperparameters with the best average performance.

Our goal was to examine the test accuracy of the model as a function of the training batch size.

## 4. Simulation Results and Discussion

In this section, we have shown simulation results where we could identify good use-cases for specific computational limitations. In these use-cases, the limitations match a task's preferred inductive bias. We further observed that, for other tasks, the same biases may well lead to a deterioration in performance. We emphasize that we did not perform exhaustive architecture searches for a given task but conversely performed a constraint search for an architecture and application that leads to an improvement over a baseline.

### 4.1. Sparse Connectivity With Fixed Weights

In this subsection, we began from a particular hardware constraint and tried to find a suitable application for it, following section 3.1 (step 1): we assumed we had developed hardware that would allow us to cheaply multiply vectors with a fixed, uniformly random matrix.

**Figure 2:**
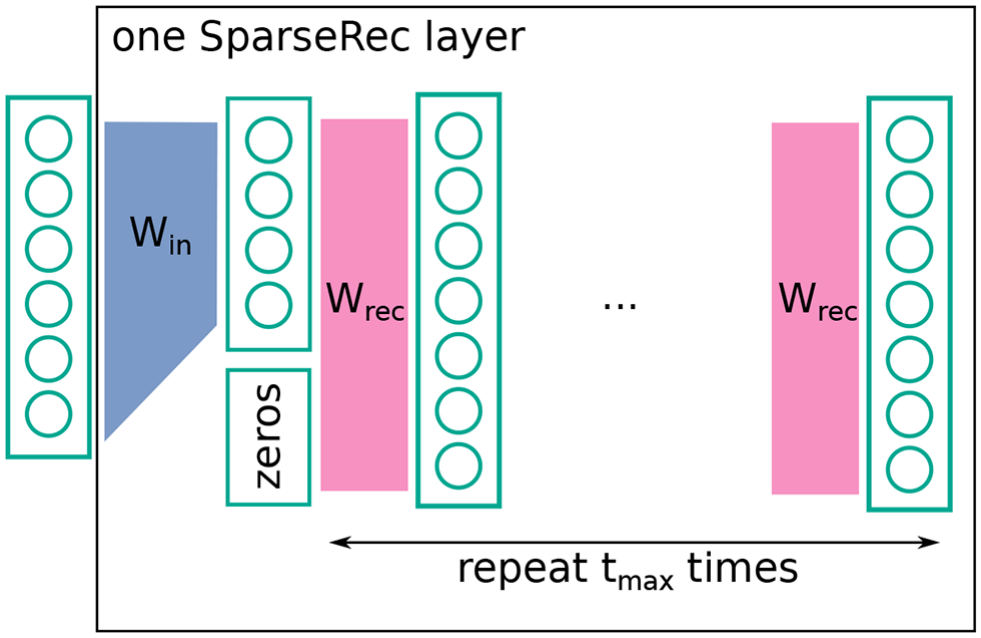
Schematic description of a sparseRec layer. *W*_*in*_ is learned, *W*_*rec*_ is fixed and random. At each layer, a non-linearity is applied. See also Equation (5).

Next, we defined an architecture (step two in section 3.1). The architecture we considered could be succinctly described as a deep echo-state network (ESN) (Gallicchio and Micheli, 2016) with trained feed-forward weights or alternatively as a set of sparsely connected feed-forward layers, with fixed random recurrent connections within each layer (see Figure 2 for a visual explanation). As given in the previous section, formulaically we proposed a neural network layer, termed the sparseRec-layer, with the following recurrent definition, given input $\vec{x}$:

$$\begin{array}{l} {\vec{y}}_{0}=W_{in}\vec{x}\\ {\vec{y}}_{t}=f\left({\vec{y}}_{0}^{*}+W_{rec}{\vec{y}}_{t-1}\right) \end{array}$$

where *W*_*in*_ is a learned input *n* × *k* matrix, and *W*_*rec*_ is a fixed, randomly drawn recurrent *m* × *m* connection matrix. We chose *m* > *k* and denoted $s=\frac{k}{m}$ as sparsity. ${\vec{y}}_{0}^{*}$ is ${\vec{y}}_{0}$ zero-padded from length *k* to *m*. *f*(·) was an activation function. When computing the output of such a layer, we applied this recurrent definition up to *t*_max_ while keeping the input fixed.

The intuition behind a sparseRec-layer, is that we want a high number of linearly independent activations in each layer. Simultaneously, we wanted to keep the number of adjustable weights small (for regularization and simpler hardware implementation) compared to the number of fixed weights. From an implementation perspective, this architecture is interesting for some of the reasons that also make ESN and Extreme learning machines (ELM) (Huang et al., 2004) appealing to hardware designers: the use of mostly fixed weights (that do not need an updating mechanism) and the recurrent network structure (that reduces information transport in comparison to a feed-forward structure). Since we added trained feed-forward weights, we required that a product of an error vector with the transpose weight matrix could also be performed for the purpose of error back-propagation (in contrast to a standard deep-esn). Crossbar-arrays (Steinbuch, 1961) are a well-known example of a kind of architecture that can support such operations.

As a “naive” first benchmark, we used MNIST (LeCun et al., 1998) and compared fully-connected networks to the proposed sparse networks with fixed random recurrent weights in each layer, as a function of number of free parameters (see Figure 4). We found a gradual degradation of the performance as the number of free parameters decreases. This is not surprising: we are not aware of any reason to expect that sparsity should improve performance for this task. Indeed, the sparse models do not exceed the performance of the dense model.

**Figure 3:**
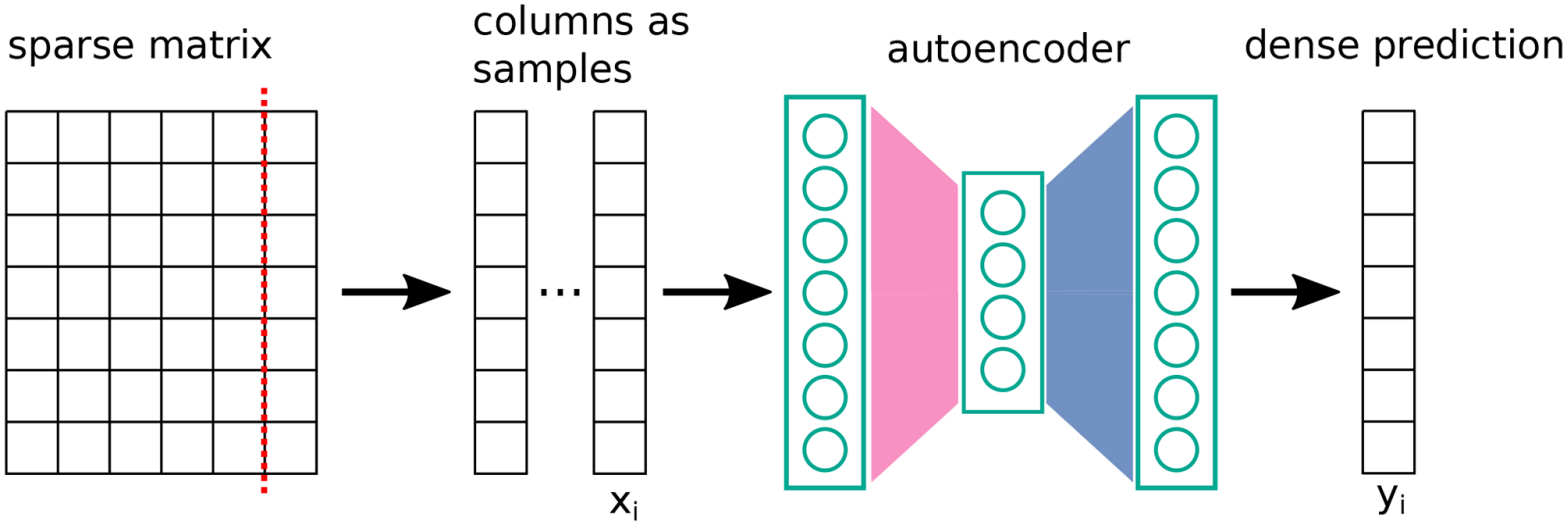
Schematic setup of the Autorec method (Sedhain et al., 2015) for predicting entries of a sparsely known matrix. The cost function is a squared error $\underset{i}{\sum }{\left(x_{i}-y_{i}\right)}^{2}$ on known entries (with regularization).

In contrast, it has been observed that sparsified networks can show improved performance in collaborative filtering settings (Muller et al., 2018) (step 3 in section 3.1). In the spirit of bias matching, we investigated whether our given sparse architecture would improve over the fully-connected baseline in this task. The setup followed (Sedhain et al., 2015) (see Figure 3). The goal was to regress missing entries of a large, sparsely known matrix given in MovieLens-1M (Harper and Konstan, 2015). To achieve this, the matrix was cut into columns or rows. Each column was treated as a sample. An autoencoder was trained to reconstruct columns, where the cost is the squared error for known entries and zero otherwise, in combination with L2-regularization. Training was performed by a gradient-descent variant, namely, L-BFGS (Zhu et al., 1997). We used the same network of Sedhain et al. (2015) as a baseline, and, for comparison, we replaced the hidden layer with the layer given in Equation (5).

Figure 5 shows that, this collaborative filtering setting, the constraint that degraded performance for the MNIST dataset, improves the performance over the fully connected baseline at a given number of free parameters. The performance also does not significantly change when decreasing the number of parameters by sparsifying in the proposed way but decreases significantly when the hidden layer is made smaller (to reach the same number of free parameters). We further found that additional network depth explains this in part by comparison to an architecture with an equal number of parameters and two hidden layers. Overall, this suggests that the constraint matches well the inductive bias required to generalize on this task.

**Figure 4:**
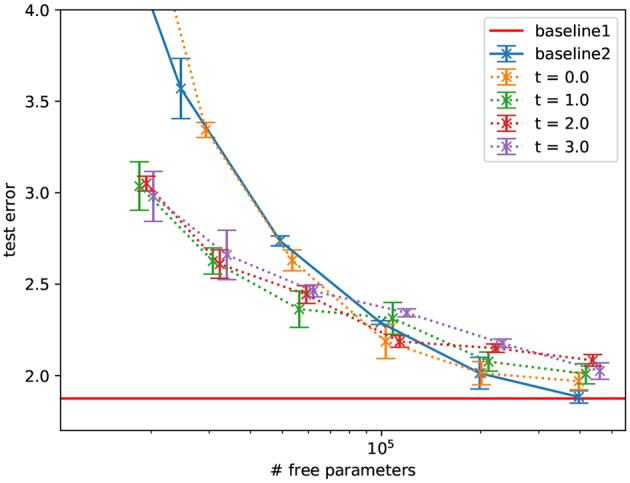
Performance of MLPs with a hidden sparseRec-layers classifying MNIST as a function of sparsity compared against two standard MLP baselines, baseline1 shows the performance at 400,000 parameters, baseline2 has a varying hidden layer size. Errorbars show the standard error. The sparse models do not exceed the performance of the dense model.

**Figure 5:**
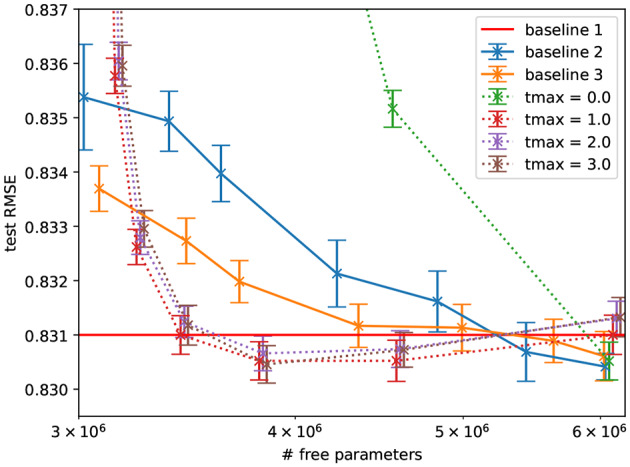
Performance of autoencoders with a hidden sparseRec-layer regressing ratings in ML1M as a function of sparsity (plotted as number of free parameters) compared against a standard autoencoder baseline. Errorbars show the standard error. Some sparse models exceed the performance of the baseline model with the same number of free parameters significantly and exceed (though not significantly) the performance of the dense model with more parameters. Baseline1: Performance reported in Sedhain et al. (2015) with 6M parameters, baseline2: our implementation of the same model with a single hidden layer of varying size, and baseline 3: with two hidden layers of varying size. Note that at 6M parameters the tmax = 0.0 model is equivalent to baseline2.

Furthermore, we found that applying the fixed, random matrix more than once does not improve the performance significantly (*t*_max_ = 1 is as good as *t*_max_ > 1). This means that our final layer architecture could be described as a learned input matrix, followed by a fixed, random matrix; in spirit, this is closer to an ELM than an ESN.

### 4.2. Batch-Size Regularization in Low-Rank Matrix Approximation

As a second example (step 1 in section 3.1), we considered a “sparse vector”-“dense matrix” multiplier where the input data vector is binary, $\vec{x}\in {\left\{0,1\right\}}^{n}$, and changes to matrix entries must occur in place. An example of such a system would be a spiking neural network with synapses implemented by a cross-bar array in the vein of Gokmen and Vlasov (2016). The key constraint we considered here is that such systems usually have difficulties aggregating gradients over multiple samples (parallelization occurs across the weight-array instead of across mini-batches).

As a computational architecture (step 2 in section 3.1), we chose the Factorization Machine (FM) (Rendle, 2010). Given a sparse sample of the entries of a matrix, we wished to regress unknown entries. As a formula, the prediction for an entry *r* of the matrix, given the concatenated one-hot encoded row and column indices *x*, is

(7) $$\begin{array}{r} r=b+\underset{i}{\sum }x_{i}w_{i}+0.5\underset{j}{\sum }\left({\left(\underset{i}{\sum }v_{ij}x_{i}\right)}^{2}-\underset{i}{\sum }v_{ij}^{2}x_{i}^{2}\right). \end{array}$$

**Figure 6:**
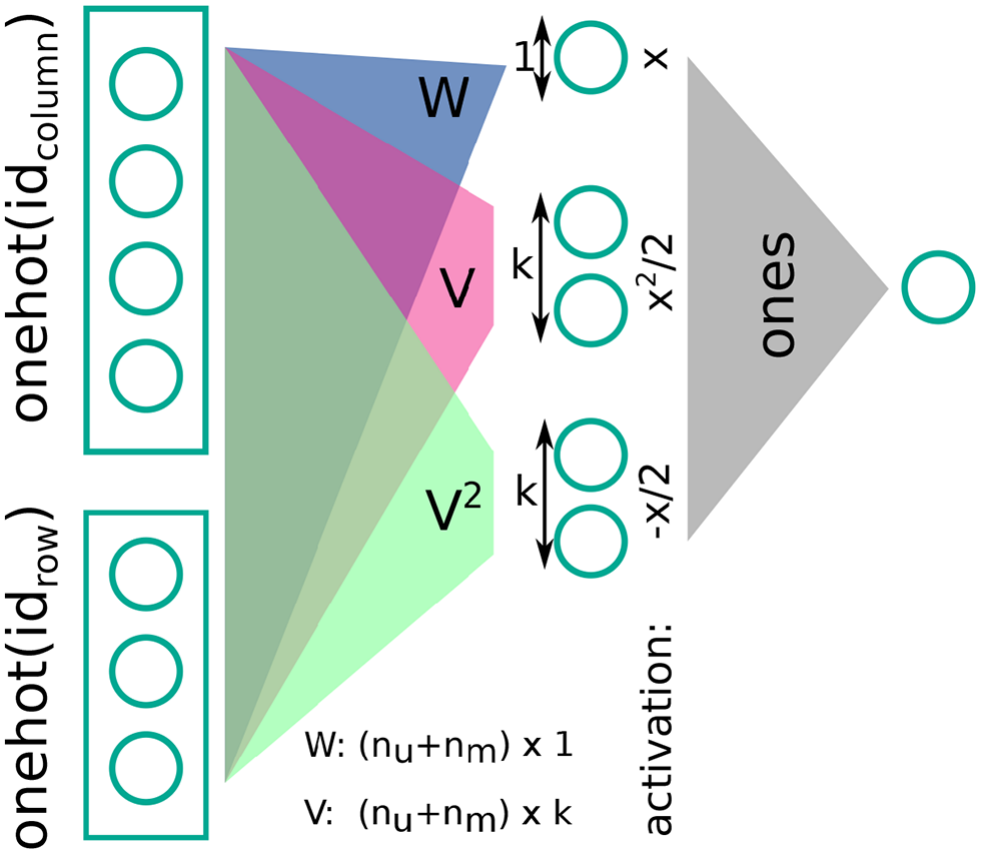
Sparse rank-k matrix decomposition (or a Factorization Machine) as a (spiking) neural network.

From a neural network perspective, we can describe the setup as follows (see Figure 6 for further details): we gave as input to three fully-connected layers the one-hot encoded row and column indices as a concatenated vector; two of these layers have a linear, the other a quadratic activation function, and their weights *W, V*_1_, *V*_2_ are tied such that $V_{1}=V_{2}^{2}$. The weights have sizes *W*:(*n*_*c*_ + *n*_*r*_) × 1 and *V*:(*n*_*c*_ + *n*_*r*_) × *k*, where *n*_*c*_, *n*_*r*_ are the number of columns and rows, respectively. The three hidden layers are read out by a dense layer of size one with fixed weights of plus one (i.e., they are summed). The output of this dense layer is the prediction for the rating. Training is performed by gradient-descent with L2-regularization.

We further note that Figure 6 makes it evident that FMs are closely related to spiking neural networks in the sense that their central operation is a sparse vector-matrix multiplication. In addition, $\vec{x}\in {\left\{0,1\right\}}^{n}$ has a clear interpretation for FMs: In this case, the FM solves a low-rank factorization problem (Rendle, 2010).

A key area of application of FMs are collaborative filtering tasks. We therefore considered low-rank matrix factorization of MovieLens-1M as a test application with the inductive bias of small training batch sizes (step 3 in section 3.1).

**Figure 7:**
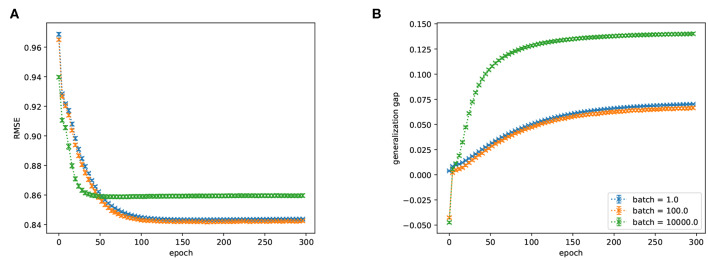
**(A)** RMSE (lower is better) and **(B)** generalization gap (difference between train and test performance, lower is better) of a FM trained on ML1M as a function of training epoch for various batch sizes. There is a significant preference for smaller batch sizes. Batch-sizes 1 and 100 are not significantly different.

We plotted the performance on a validation set of this network as a function of the mini-batch size during training (Figure 7). We found that increasing batch-sizes reduce the performance of the network (but note that it is possible that means of regularization other than the ones we tested allow for the use of larger batch-sizes). This indicates that an interesting area of application for weight-parallel spiking neural network accelerators are FMs because they can give a (weight-wise) parallelization speed-up without the performance degradation associated with large batch-sizes.

We note that the beneficial effect of using SGD with small batch-sizes has been observed in other applications as well (as mentioned in section 2.6, e.g., Wilson and Martinez, 2003).

## 5. Conclusions

When one approximates a machine learning model efficiently, assuming some hardware constraints, the usefulness of these constraints for generalization is worth careful consideration. In other words, hardware constraints must match inductive biases. Such a match can lead to highly efficient and well-performing systems. For example, when designing a neuromorphic chip to analyze speech signals, it does not need to support fast state changes in the hidden neurons (see section 2.3), and building accelerators for collaborative filtering exploiting sparsity could be very relevant (see section 4.1).

Similarly, avoidance of an inappropriate bias can also be crucial, as demonstrated by the Shuffle-Net (Zhang et al., 2018), where a factorization of the model into independent subnetworks is avoided by random shuffling of sparsely connected channels.

Recently, the question has arisen as to whether, in machine learning research, the most successful approach is to look for ways to apply more computational power to a problem rather than finding better designed solutions (Sutton, 2019). Through the many examples of “bias-matching” we have reported in this paper, we support the contrary notion that finding low-level improvements (through hardware constraints) that synergize with the problems one is trying to solve (through inductive biases) is a kind of thoughtful problem solving that can be crucial in the development of competitive machine learning systems.

The embodiment of inductive biases as hardware constraints also implies a caveat for the evaluation of neuromorphic architectures: if an architecture aims to be general purpose, it is important to benchmark it on a variety of tasks; otherwise, it may be the case that the chosen benchmarks benefit from inductive biases embodied by the constraints of the given architecture.

In this paper, we discussed several examples from the literature where such a match is given. Furthermore we applied the idea of bias matching to a novel network architecture that can make use of fixed, random weights, and found that its sparse structure leads to improved performance over a dense baseline on a benchmark for which sparsity has been shown to be useful previously.

## Data Availability Statement

The datasets generated for this study are available on request to the corresponding author.

## Author Contributions

All authors listed have made a substantial, direct and intellectual contribution to the work, and approved it for publication.

## Conflict of Interest

All authors were employed by the company IBM.
